# *Ex vivo* nephron-sparing surgery and kidney autotransplantation for renal tumors

**DOI:** 10.1093/jscr/rjab004

**Published:** 2021-02-16

**Authors:** Francesco Crafa, Amalia Rosaria Rita Rossetti, Augusto Striano, Mario Baiamonte, Francesco Esposito

**Affiliations:** Department of Oncological and General Surgery, S.G. Moscati Hospital, Avellino, Italy; Department of Oncological and General Surgery, S.G. Moscati Hospital, Avellino, Italy; Department of Oncological and General Surgery, S.G. Moscati Hospital, Avellino, Italy; Department of General and Emergency Surgery, Civico Hospital, Palermo, Italy; Department of Digestive and Oncological Surgery, Grand Hopital de l’Est Francilien, Jossigny, France

## Abstract

The nephron-sparing techniques allow the excision of kidney tumors preserving renal function and ensuring adequate oncological results. We describe a case of a 77-year-old patient who underwent an *ex vivo* partial nephrectomy with orthotopic autotransplantation for kidney cancer. The postoperative course was marked by bleeding which required radiological embolization. Postoperative dialysis was required for about 1 month. The anatomopathological examination showed a clear cell carcinoma staged pT1b, pNX, R0. At 2 years follow-up, no recurrence was detected with a complete renal function restoration. Our experience shows that *ex vivo* nephron-sparing surgery with autotransplantation is a good alternative to total nephrectomy in the case of voluminous or perihilar tumors. Considering the high morbidity of this procedure, it should be only performed in specialized centers.

## INTRODUCTION

The nephron-sparing techniques allow the excision of the lesion, preserving renal function and ensuring adequate oncological results [1]. This procedure is recommended in patients with a solitary kidney, voluminous sized-masses, centro-renal location and when the *in vivo* partial nephrectomy (PN) is difficult to achieve. Hardy *et al*. in 1963, described for the first time the treatment of a severe traumatic lesion of the ureter treated with explantation, repair and autotransplantation [2]. Since then, this procedure has been used extensively to repair ureter injuries, renal artery aneurysms and much more rarely for kidney cancers [3].

In the present study, we report our experience with *ex-vivo* PN and kidney autotransplantation for renal tumors.

## CASE REPORT

A 77-year-old woman was admitted for an asymptomatic mass of the left kidney. The patient was overweight (BMI: 27.3 kg/m^2^), had arterial hypertension and non-insulin-dependent diabetes mellitus. The creatinine value and preoperative glomerular filtration rate were within normal limits.

**Figure 1:**
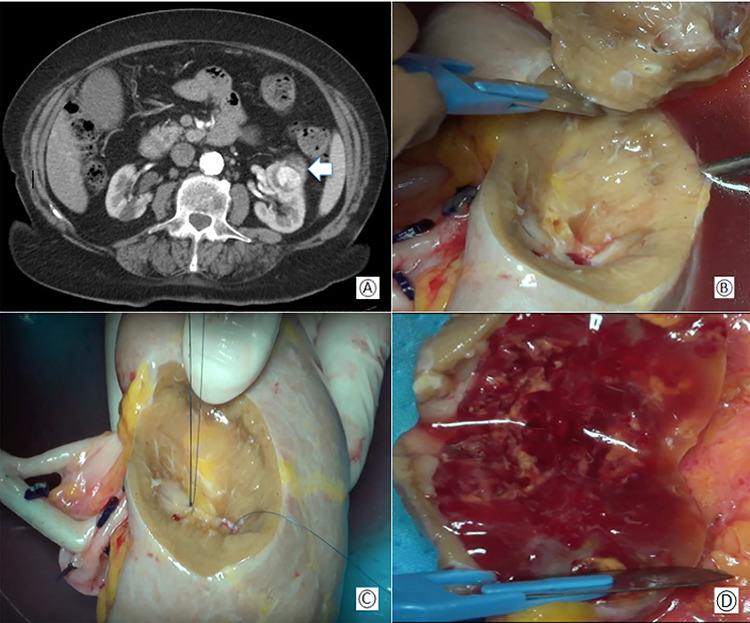
(**A**) Preoperative computed tomography: the white arrow shows the renal mass; (**B**) removal of the tumor during bench surgery; (**C**) suture of the renal calices during bench surgery; (**D**) macroscopic examination of the surgical piece.

CT-scan showed a mass of 50 × 30 × 40 mm diameter in the anterior and middle region of the left kidney involving the medium calyceal group (Fig. 1A). Due to the size of the tumor and its central location, an *ex-vivo* PN and kidney autotransplantation was planned.

Xifo-pubic incision was performed. The left colon and spleen were mobilized exposing the left kidney. The ureter was identified at the level of the iliac vessels and the homolateral gonadal vessels were sectioned. The renal hilum was exposed and then, renal artery and vein were clamped and dissected. The kidney was immediately immersed in ice-cold serum and perfused with 4^°^C hypertonic citrate adenine solution through the renal artery.

**Figure 2:**
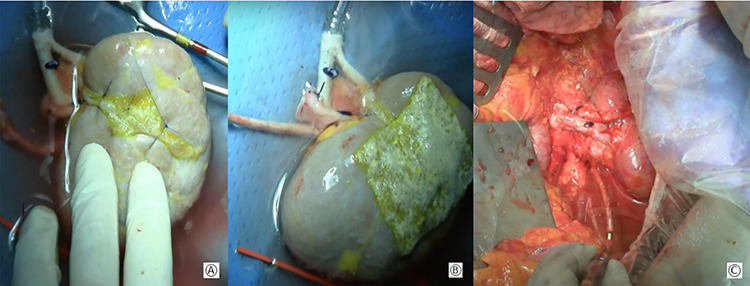
(**A**) Approximation of the margins of the section with interposition of a hemostatic sponge; (**B**) kidney at the end of the bench surgery; (**C**) intraoperative view after vascular anastomoses.

The renal capsule was incised and the tumor was resected with at least 1 cm margin of healthy parenchyma, with microscopic frozen section examination negative for neoplastic residue (Fig 1B–D). The sectioned vessels were tied with polypropylene 6–0 (Ethicon®), while the renal calices and the pelvis were sutured with a polypropylene 7–0 running suture (Ethicon®) (Fig. 2A and B). The kidney was repositioned in its natural location (Fig. 2C). End-to-end artery anastomosis with polypropylene 7–0 separate stitches and end-to-end vein anastomosis with two running sutures in polypropylene 6–0 were performed. Double J silicone catheter (Coloplast, Bologna) was inserted and the ureteral stumps were anastomosed with PDS 6–0 (Ethicon®). The hemostasis on the residual renal parenchyma was completed with polypropylene 6–0 separate stitches and with a hemostatic sponge (TachoSil®- Nycomed, Konstanz, Germany). Two drains were placed anteriorly and posteriorly to the surgical site.

The total duration of the procedure was 4 h with a total ischemia time of 2 h. The mean estimated blood loss was 120 ml. No intraoperative complications occurred. The immediate postoperative course was marked by an increase of creatinine value and diuresis contraction for which the patient underwent dialysis for 1 month. On the 10th postoperative day, the patient showed signs of active bleeding, confirmed by a CT-scan with the presence of a lower polar artery pseudoaneurysm resolved by radiological embolization.

The subsequent course was uneventful and the drains were removed on the 12th postoperative day.

The patient was discharged on the 20th postoperative day with a progressive renal function improvement. Dialysis was needed for about a month and was subsequently stopped. The double J silicone catheter was removed 5 weeks later.

The anatomopathological examination showed a clear cell carcinoma, with a stage II Fuhrman grading. The pathological tumor stage was pT1b, pNX, R0.

At 24 months follow-up, no evidence of local or distance recurrence was detected and renal function was normal.

## DISCUSSION

The main indications for *ex vivo* nephron-sparing surgery and kidney autotransplantation are centrally or perihilar located tumors, when PN *in situ* is risky due to the impossibility of guaranteeing free surgical margins. In addition, it is the only valid option for patients who have an acquired or functional solitary kidney, as Ono *et al*. [4] showed in their case report.

This technique is also an option for multifocal or bilateral tumors; Abraham *et al*. [5] described a case of a young patient with giant bilateral angiomyolipoma who was treated with a bilateral open nephrectomy, *ex-vivo* PN and kidney autotransplantation in two separate sessions.

Concerning surgical approach, laparoscopic and open PN have the same indications with similar oncological outcomes and intraoperative complication rates when performed in high experience centers [6, 7]. According to our expertise, we decided to perform an open surgical approach.

Regarding the anatomical kidney implantation, it is usually placed in the iliac fossa with anastomosis between the renal and iliac vessels [6, 8]. Only Kulisa *et al*. [7] performed an orthotopic transplant, like in our patient. In the case of autotransplantation for ureteral injuries or vascular aneurysms, implantation in a heterotopic site is required. In the case of cancerous lesions, implantation at the anatomical site may be a viable alternative.

Acute vascular bleeding and urinary fistulas are the most frequent complications [7, 9]. In our case, the patient developed an artery pseudoaneurysm and an active bleeding that required an interventional radiology procedure. Kulisa *et al*. [7] reported two cases of vascular thrombosis requiring surgery and removal of the transplanted kidney.

Functionally, acute tubular necrosis was the main cause of dialysis and it may be related to the time of cold ischemia [7].

In conclusion, *ex vivo* PN with autotransplantation is a good alternative to total nephrectomy in the case of bulky and perihilar tumors. Considering the high morbidity of this procedure, it should be performed in expert centers.
